# Prophets at Home

**Published:** 1862-12

**Authors:** 


### PROPHETS AT HOME.

THAT best and wisest of all books contains the sentiment that, "A prophet is not without honor, save in his own country, and in his own house." Home product is not valued as highly as that which comes from abroad. The *Home Journal*, always well edited, has grown greatly in its influence within a year or two, by its increased adaptability to the times, as to the substantial utility of its articles, especially in connection with the war. The issue for November 1st contains two columns closely printed, in small type, in reference to the influence which living in the country has upon the health of those who do business in town, especially when they go in and out by rail. Nearly six years ago we wrote a few pages on the subject, which happened to be among the few articles of our JOURNAL which not one of our exchanges thought it worth while to copy. But now, when mainly the same sentiments and the same reasons for them, appear in a foreign periodical, under so distinguished a name as that of Dr. Forbes Winslow, one of the most able of British medical writers, corroborated by two other eminent names, Dr. C. J. B. Williams, and Dr. Angus Smith, they are reproduced at great length, as if the subject itself and the views therein were new. We therefore reproduce our own article, first published in March, 1857, because it is more directly to the point, in fewer words, and more easily comprehended by that great mass of Americans, who must do two things at a time, (so fast are they,) who can give only that time to reading which is passed in riding from one place to another, and more, who demand that what they read should be so plain that it can be understood at a glance, without any more intellectual effort than is required to comprehend a sum in simple subtraction, such as four from two leaves six. But as the JOURNAL now circulates among outside barbarians, as in the Sandwich Islands, Canada, New-Brunswick, Nova Scotia, Prince Edward's Island, London, Paris, Constantinople, and even in the Holy City itself, it may be well enough to make a note of explanation of that little mathematical problem, by the statement of a fact, personal to ourself. When we were married, some years ago, two of us sat down to table, but there are now six of us; so "we two," and four children — the four from the two (as far as we know and believe)—make six; *quod erat demonstrandum.*

And now for our article, written six years ago, to show that, *ordinarily*, health is not promoted, but is endangered, and often lost, by living in the country and doing business in town; and that there is an actual loss of quietude of mind and bodily comfort may very readily be inferred from the fact that most of those who go to the country, soon get tired of it, and are willing, in multitudes of cases, to sell out at a loss, in order to get back to town again. It seems to us that even on the beautiful banks of the Hudson, three persons out of every four, engaged in business in the city, are very willing, even eager to "sell out."

